# Updated Information on Risk Factors for Lung Cancer: Findings from the JACC Study

**DOI:** 10.2188/jea.15.S134

**Published:** 2005-08-18

**Authors:** Kenji Wakai, Masahiko Ando, Kotaro Ozasa, Yoshinori Ito, Koji Suzuki, Yoshikazu Nishino, Shin-ichi Kuriyama, Nao Seki, Takaaki Kondo, Yoshiyuki Watanabe, Yoshiyuki Ohno, Akiko Tamakoshi

**Affiliations:** 1 Division of Epidemiology and Prevention, Aichi Cancer Center Research Institute.; 2 Kyoto University Center for Student Health.; 3 Department of Epidemiology for Community Health and Medicine, Kyoto Prefectural University of Medicine Graduate School of Medical Science.; 4 Department of Public Health, Fujita Health University School of Health Sciences.; 5 Division of Epidemiology, Department of Public Health and Forensic Medicine, Tohoku University Graduate School of Medicine.; 6 Department of Infectious Disease Control and International Medicine, Niigata University Graduate School of Medical and Dental Science.; 7 Department of Medical Technology, Nagoya University School of Health Sciences.; 8 Department of Preventive Medicine/Biostatistics and Medical Decision Making, Nagoya University Graduate School of Medicine.

**Keywords:** Lung Neoplasms, Smoking, Diet, Carotenoids, Insulin-Like Growth Factor I, Insulin-Like Growth Factor II

## Abstract

BACKGROUND: In Japan, lung cancer is the top cause of cancer death in men and the third leading cause in women. Updated information on risk factors for lung cancer, therefore, is of great importance. The Japan Collaborative Cohort Study, a large prospective study started in 1988, has provided such information.

METHODS: We reviewed published findings for lung cancer from the study. The endpoint was death from this cancer.

RESULTS: The major findings were as follows. (1) The relative risks in current smokers versus nonsmokers were 4.46 in men and 3.58 in women. (2) Cigarette smoking accounted for 67.0% of lung cancer deaths in men and only 14.6% in women. (3) More than 15 years of smoking cessation may be required to decrease the risk of lung cancer to the level of never smokers. (4) A reduced risk was associated with frequent intake of green-leafy vegetables and fruit in men but not in women. These foods seemed to decrease the risk in male current or former smokers more than in female nonsmokers. (5) Serum levels of *α*- and *β*-carotenes, *β*-cryptoxanthin, and lycopene were inversely correlated with the risk in men. (6) In a preliminary study, serum 8-hydroxy-deoxyguanosine was higher in current smokers than in nonsmokers.

CONCLUSIONS: The relative and attributable risks of smoking were smaller in Japan than in Western countries. In addition to smoking habits, therefore, we must pay attention to other risk factors for lung cancer or factors that modify the adverse effects of smoking including dietary factors.

In Japan, lung cancer is the top cause of cancer death in men and the third leading cause after stomach and colorectal cancers in women in 2000.^1^ In both genders, the age-adjusted mortality rate of this cancer had been increasing until around 1995. Although the rate has since leveled off,^1^ it can increase again because of the high consumption of tobacco among baby boomers after World War II.^2^

Updated information on risk factors for lung cancer in Japan is thus of great importance, considering the widespread impact of this malignancy. Nevertheless, the risk factors other than smoking have never been examined in a large prospective study in Japan since the Six-Prefecture Cohort Study initiated by Hirayama et al. in 1965.^3^

We therefore have elucidated risk and protective factors for this cancer in the Japan Collaborative Cohort Study (JACC Study) for Evaluation of Cancer Risk sponsored by the Ministry of Education, Science, Sports and Culture of Japan (Monbusho). The baseline survey of this study started in 1988,^4^ and the study has provided up-to-date data for primary prevention of cancer in this country. In this article, we briefly review published findings for lung cancer from the study.^5^^-^^11^ The endpoint for all the studies on lung cancer was death from this cancer.

## Smoking

The relative risk of lung cancer in current smokers versus never smokers is reported to be much lower in Japan than in Western countries.^12^ One of the possible explanations for this gap is the tobacco shortage during and immediately after World War II in Japan. If this is indeed the case, the relative risk would increase in recent studies due to the recovery of tobacco production in postwar Japan along with the declining effects of the insufficiency.

Ando et al.,^8^ however, reported that the relative risk in male current smokers was 4.46, which was quite comparable with the figure (4.5) in the Japan Public Health Center-based prospective study (JPHC Study), another population-based cohort study started in 1990.^12^ These relative risks were almost the same as that in the Six-Prefecture Cohort Study (4.45) started in 1960s.^3^ The very similar risks between the three cohort studies suggest that either the effect of the wartime tobacco shortage still remains in Japan or that the comparatively lower relative risk cannot be ascribable to the insufficiency. In contrast, the relative risks in female current smokers were 3.58 in the JACC Study and 4.2 in the JPHC Study,^12^ respectively, which were greater than that in the Six-Prefecture Cohort Study (2.34).^3^ The increase in the risk may be attributable to the increasing intensity of female smoking.

In the JACC Study, cigarette smoking accounted for 67.0% of lung cancer deaths in men and only 14.6% in women.^8^ This may imply the importance of risk factors other than smoking habits or factors that modify the risk of smoking.

To assess the decrease in risk of lung cancer after quitting smoking is indispensable to estimate the effect of smoking cessation program. Wakai et al.^6^ addressed this issue using the dataset from the JACC Study. They reported that the relative risk, compared to nonsmokers, was 5.16 for current smokers, and 4.84, 3.19, 2.03, 1.29, and 0.99 for ex-smokers who had quit smoking 0-4, 5-9, 10-14, 15-19, and 20+ years before baseline, respectively. This finding means that more than 15 years of cessation may be required to decrease the risk of lung cancer to the level of never smokers. The authors also pointed out that the reduction in the mortality rate of lung cancer was larger in smokers who stopped smoking at younger ages.^6^

Because long periods are needed to cancel the risk in current smokers, never starting smoking at all costs must be encouraged as well as stopping smoking. Ando and colleagues estimated that 31.2% of lung cancer deaths in men and 4.7% in women could be prevented by smoking cessation according to their unique analysis using proportional hazards models.^8^ They computed the expected number of deaths from lung cancer assuming all the current smokers at baseline became ex-smokers at that time and compared it with the number in the observed situation. The difference between the two figures was regarded as the number of lung cancer deaths preventable if all the smokers had had successfully stopped smoking at baseline. When comparing the estimated percentages with the attributable risk percent mentioned above (67.0% in men and 14.6% in women), smoking cessation programs alone would at best decrease lung cancer deaths due to smoking by half or less, and the remainder may be preventable only by discouraging commencement of smoking.^8^

## Dietary Factors

Vegetables and fruit have long been reported to reduce risk of lung cancer in case-control or cohort studies.^13^ This view, however, has recently been challenged by the failure of *β*-carotene supplementation to prevent lung cancer^14^^-^^16^ and by some null results in recent large prospective studies.^17^ Although the Six-Prefecture Cohort Study contributed to form the hypothesis,^3^ the role of diet in the etiology of lung cancer should also be reappraised in Japan to obtain updated information for public health.

In the JACC Study, Ozasa et al.^5^ reported a reduced risk of lung cancer death in relation to frequent intake of green-leafy vegetables, oranges, and fruits other than oranges in men but not in women. In men, a 20-30% decrease in risk was found in the highest category of intake frequency compared with the lowest. These foods seemed to decrease the risk in male current or former smokers more than in female nonsmokers. This may indicate that the protective effects of green vegetables and fruit are stronger in men or in smoking-related lung cancer such as squamous or small cell carcinoma. Unfortunately, it was difficult to analyze the data by cell type because cell type was unknown for a substantial proportion of lung cancer cases. Female never smokers who frequently consumed ham and sausages, liver, or fried foods were at an enhanced risk; the hazard ratio for intake of ≥ 3-4 times/week versus ≤ 1-2 times/month ranged from 1.91 to 2.29. This finding may be in line with the hypothesis that high fat consumption increases the risk of lung cancer, especially that of adenocarcinoma.

In addition to the difference in genetic and environmental backgrounds between study populations, some methodological issues may explain the inconsistent findings on the association of vegetable and fruit consumption with lung cancer risk. Ozasa et al.^5^ estimated the relative risk after adjustment for smoking with classifying ex- and current smokers into very detailed strata. However, possible confounding by physical activity, use of vitamin supplements, fat intake, or health-consciousness^17^ was not controlled. They related single food items, instead of vegetables and/or fruit as a whole,^17^ to the risk of lung cancer death. Additional analyses of the JACC Study data considering these issues would further provide useful information.

Ito and coworkers^9^^,^^10^ examined the association of serum carotenoids with the risk of lung cancer death in nested case-control studies among participants in the JACC Study who donated blood samples at baseline, and found that serum levels of several carotenoids were inversely correlated with the risk in men. The dose-response relationship was most clear for *α*- and *β*-carotenes; the multivariate-adjusted odds ratios (ORs) across quartiles were 1.00, 0.88, 0.60, and 0.40 for *α*-carotenes (trend p = 0.013) and 1.00, 0.78, 0.71, and 0.23 for *β*-carotene (trend p = 0.003) in the follow-up through 1999.^10^ Such inverse associations with a significant trend in risk were observed for *β*-cryptoxanthin and lycopene. Although a sufficient sample size was not available for women, a decreasing trend in risk was suggested with an increasing level of serum total carotenoids (trend p = 0.077).

When considering these findings with the results based on intake frequency of vegetables and fruit,^5^ keeping high blood levels of carotenoids by consuming green vegetables and fruit instead of taking *β*-carotene supplements may remain beneficial in helping to prevent male or smoking-related lung cancer in Japan. Because blood concentrations of carotenoids are lower in smokers than in nonsmokers, as Suzuki et al.^11^ confirmed, smokers could benefit more from consuming green vegetables and fruit. Further investigations are warranted for the possible risk-decreasing effects of *β*-cryptoxanthin because not dietary or blood *β*-carotene but *β*-cryptoxanthin was negatively associated with the risk of lung cancer in some cohort studies,^18^ and *β*-cryptoxanthin may confound the protective effect of *β*-carotene.

## Serum Markers Other than Carotenoids

Insulin-like growth factors (IGFs) and IGF-binding proteins (IGFBPs) have been investigated in relation to the risk of various cancers.^7^ IGF-I and IGF-II may promote the development of cancer by stimulating cell proliferation. IGFBP-3, the principal blood IGFBP, binds most of the IGFs in blood, thereby limiting the biological activity of IGFs. IGFBP-3 also inhibits cellular proliferation and induces apoptosis by binding to IGFBP-3 receptors. Epidemiological data on IGFs and IGFBP-3 for lung cancer, however, remain insufficient compared with those for breast, colorectal, and prostate cancers.^19^

In the JACC Study, serum IGF-I, IGF-II, and IGFBP-3 were determined for incident cases of cancer and all dead cases and their controls matched for age, gender, and study area. Wakai et al.^7^ analyzed the dataset for lung cancer and found smaller ORs with higher levels of IGF-II and IGFBP-3; the ORs over quartiles were 1.00, 0.41, 0.47, and 0.67 for IGF-II (trend p = 0.018) and 1.00, 0.55, 0.54, and 0.67 for IGFBP-3 (trend p = 0.037). The risk was increased in the highest quartile of IGF-I after controlling for IGFBP-3 (OR, 1.74). The findings for IGF-I and IGFBP-3 appeared to support the working hypothesis while the inverse association of IGF-II did not. Further studies on IGF-II, however, would be required since some have reported a decreased risk of prostate^20^ and breast^21^ cancers associated with higher blood IGF-II.

Smoking is known to generate reactive oxygen species (ROS) *in vivo*. DNA damage induced by ROS is quickly repaired by exonucleases, and the isolated 8-hydroxy-deoxyguanosine (8-OHdG) has been studied as a biomarker for oxidative DNA damage. Suzuki et al.,^11^ in a preliminary study on determination of oxidation or anti-oxidation markers in circulation, found that the serum 8-OHdG level was much higher in male current smokers than in nonsmokers; the median was 0.30 ng/mL and 0.07 ng/mL, respectively. We attempted but failed to correlate serum levels of 8-OHdG with the risk of lung cancer in the JACC Study. The reproducibility of the measurement of serum 8-OHdG might be still too low^11^ to detect a small difference in the level between lung cancer cases and controls in a nested case-control study.

The major findings mentioned above are summarized in Table 1 with some remarks. In summary, the highest priority must be given to the antismoking campaigns or education in the prevention of lung cancer. However, the relative and attributable risks of smoking were rather smaller in Japan than in Western countries. Therefore, we must also pay attention to risk factors other than smoking habits or factors that modify the adverse effects of smoking including dietary factors. Further studies on blood risk markers for lung cancer are also warranted for the better risk assessment and the evaluation of intervention to decrease the risk.

**Table 1.** Major published findings for lung cancer from the Japan Collaborative Cohort Study.

Findings	Remarks
Smoking	
The relative risks in current smokers versus nonsmokers were 4.46 in men and 3.58 in women.^8^ Cigarette smoking accounted for 67.0% of lung cancer deaths in men and only 14.6% in women.^8^ More than 15 years of smoking cessation may be required to decrease the risk of lung cancer to the level of never smokers.^6^	The relative risk in current smokers compared with nonsmokers in large, population-based cohort studies in Japan: Six-Prefecture Cohort Study: 4.45 in men and 2.34 in women;^3^ Japan Public Health Center-based prospective study: 4.5 in men and 4.2 in women.^12^

Dietary factors	
A reduced risk was associated with frequent intake of green-leafy vegetables and fruit in men but not in women. These foods seemed to decrease the risk in male current or former smokers more than in female nonsmokers.^5^ Serum levels of *α*- and *β*-carotenes, *β*-cryptoxanthin, and lycopene were inversely correlated with the risk in men.^9^^,^^10^	Some recent large prospective studies did not show a decrease in risk associated with consumption of vegetables and fruit.^17^ Not dietary or blood *β*-carotene but *β*-cryptoxanthin was negatively associated with the risk of lung cancer in some cohort studies.^18^

Serum markers other than carotenoids	
Smaller risks were found in those with higher serum IGF-II and IGFBP-3 while the risk was increased in the highest quartile of IGF-I after controlling for IGFBP-3.^7^ Serum 8-hydroxy-deoxyguanosine was higher in current smokers than in nonsmokers (preliminary results).^11^	Some studies have reported a decreased risk of prostate^20^ and breast^21^ cancers associated with higher blood IGF-II.

IGF: insulin-like growth factor.

IGFBP: IGF-binding protein.

## MEMBER LIST OF THE JACC STUDY GROUP

The present investigators involved, with the co-authorship of this paper, in the JACC Study and their affiliations are as follows: Dr. Akiko Tamakoshi (present chairman of the study group), Nagoya University Graduate School of Medicine; Dr. Mitsuru Mori, Sapporo Medical University School of Medicine; Dr. Yutaka Motohashi, Akita University School of Medicine; Dr. Ichiro Tsuji, Tohoku University Graduate School of Medicine; Dr. Yosikazu Nakamura, Jichi Medical School; Dr. Hiroyasu Iso, Institute of Community Medicine, University of Tsukuba; Dr. Haruo Mikami, Chiba Cancer Center; Dr. Yutaka Inaba, Juntendo University School of Medicine; Dr. Yoshiharu Hoshiyama, University of Human Arts and Sciences; Dr. Hiroshi Suzuki, Niigata University School of Medicine; Dr. Hiroyuki Shimizu, Gifu University School of Medicine; Dr. Hideaki Toyoshima, Nagoya University Graduate School of Medicine; Dr. Kenji Wakai, Aichi Cancer Center Research Institute; Dr. Shinkan Tokudome, Nagoya City University Graduate School of Medical Sciences; Dr. Yoshinori Ito, Fujita Health University School of Health Sciences; Dr. Shuji Hashimoto, Fujita Health University School of Medicine; Dr. Shogo Kikuchi, Aichi Medical University School of Medicine; Dr. Akio Koizumi, Graduate School of Medicine and Faculty of Medicine, Kyoto University; Dr. Takashi Kawamura, Kyoto University Center for Student Health; Dr. Yoshiyuki Watanabe, Kyoto Prefectural University of Medicine Graduate School of Medical Science; Dr. Tsuneharu Miki, Graduate School of Medical Science, Kyoto Prefectural University of Medicine; Dr. Chigusa Date, Faculty of Human Environmental Sciences, Mukogawa Women’s University ; Dr. Kiyomi Sakata, Wakayama Medical University; Dr. Takayuki Nose, Tottori University Faculty of Medicine; Dr. Norihiko Hayakawa, Research Institute for Radiation Biology and Medicine, Hiroshima University; Dr. Takesumi Yoshimura, Fukuoka Institute of Health and Environmental Sciences; Dr. Akira Shibata, Kurume University School of Medicine; Dr. Naoyuki Okamoto, Kanagawa Cancer Center; Dr. Hideo Shio, Moriyama Municipal Hospital; Dr. Yoshiyuki Ohno, Asahi Rosai Hospital; Dr. Tomoyuki Kitagawa, Cancer Institute of the Japanese Foundation for Cancer Research; Dr. Toshio Kuroki, Gifu University; and Dr. Kazuo Tajima, Aichi Cancer Center Research Institute.
